# Indiana State Medical Society

**Published:** 1872-06

**Authors:** 


### *Indiana State Medical Society.*

The Indiana State Medical Society commenced its Twenty-second Annual Session in the lecture room of the Indiana Medical College, in Indianapolis, on Tuesday, May 21, 1872, at 10 o'clock, A. M.

Dr. H. P. Ayres, of Fort Wayne, elected President at the last annual session, assumed the duties of the Chair with the following address:

*Gentlemen of the Indiana State Medical Society*—I appreciate the honor you have conferred upon me; I ask your indulgence and assistance in the discharge of my duties.

Permit me to congratulate you on this re-union of 1872, after the incidents and changes of another year. I hope it will be one memorable for its pleasures, sociabilities and harmonies; that old friendships may be renewed and perpetuated; that new ones may be formed and cherished; but, above all, that new impulses may urge us on as medical men to accomplish the great object of our mission, viz: the health and happiness of mankind. And when we return to our homes, may we be invigorated and strengthened for the duties and labors of another year, continually looking forward with pleasure to other, and still other re-unions, while life continues. I thank you for the honor.

The Finance Committee submitted the following recommendation :

Your Committee would suggest that, in their opinion, the dues should be three dollars per member. T. M. STEVENS, *Chairman*.

On motion of Dr. J. H. Woodburn, the recommendation was adopted.

The President announced as a Committeee on Credentials, Drs. R. E. Haughton, of Richmond, I. C. Walker, of Indianapolis, and J. R. Wiest, of Richmond.

Also, as a Committee on Membership at Large, Drs. I. Casselberry, of Evansville, Wilson Hobbs, of Carthage, and J. Thompson, of Indianapolis.

The Committee on Credentials, through Dr. J. R. Wiest, submitted the following report :

The Committee on Credentials recommend the following delegates for membership in the State Medical Society :

Dr. John H. Spurrier, Rush Medical Society ; Dr. Wm. M. Hill, Mott Medical Society, Greenfield ; Dr. H. Tilson, Mott Medical Society, Greenfield ; Dr. M. M. Adams, Mott Medical Society, Greenfield ; Dr. H. C. Vincent, Dearborn County Medical Society, Lawrenceburg ; Dr. J. M. Runyan, Wabash County Medical Society, Wabash ; Dr. T. C. Kimball, Grant County Medical Society, Xenia ; Dr. J. L. Gilbert, N. E. Indiana Medical Society, Kendallville; Dr. Thomas C. VanNuys, Evansville Medical Society, Evansville ; Dr. W. R. Davidson, Evansville Medical Society, Evansville ; Dr. L. J. Woollen, Switzerland Medical Society, Moorefield ; Dr. J. S. Gregg, Allen County Medical Society, Ft. Wayne ; Dr. Jas. H. Bates, Grant County Medical Society, Jonesboro ; Dr. John H. McIntire, Wayne County Medical Society, Richmond ; Dr. Edwin Hadley, Wayne County Medical Society, Richmond ; Dr. W. C. Williams, N. E. Indiana Medical Society, Albion.

J. R. WIEST,<br>R. E. HAUGHTON, } *Committee*.<br>I. C. WALKER,

Dr. I. Casselberry submitted the following report :

The Committee on Membership at Large, respectfully recommend for admission the following persons : Dr. J. F. Mitchell, Vernon, Jennings county, Indiana ; Dr. William N. McCoy, Jeffersonville, Indiana ; Dr. J. C. Driver, Sheilville, Hamilton county, Indiana ; Dr. W. M. Glass, Sheilville, Hamilton county, Indiana ; Dr. J. G. Jones, North Vernon, Jennings county, Indiana ; Dr. Edmund D. Laughlin, Orleans, Orange county, Indiana ; Dr. John D. Simpson, Bedford, Lawrence county, Indiana ; Dr. William I. Hall, of Gessio, Vermillion county, Indiana.

I. CASSELBERRY,<br>J. THOMPSON, } *Committee*.<br>W. HOBBS,

Both reports were concurred in and the persons named elected to membership.

Dr. Green V. Woolen, Secretary, read the following report :

Your Secretary would respectfully report that he has issued the certificates to delegates and members, and notified delinquents, and received the annual dues, $3.00, from the following members since the publication of last year's Transactions :

Dr. M. G. Sherman, Michigan City ; Dr. J. A. Nesbit, Castleton ; Dr. J. A. Stillwell, Brownstown ; Dr. B. Ward, Indianapolis ; Dr. R. D. Mauzy, Rushville ; Dr. W. L. Haymond, Monticello ; Dr. H. F. Barnes, Louisville, Ky.; Dr. J. M. Jossie, Ft. Wayne ; Dr. M. H. Harding, Lawrenceburg ; Dr. G. W. Luntz, Millhausen.

Total amount collected from dues, $593, which has been paid to the Treasurer.

G. V. WOOLEN, *Secretary*.

Dr. James H. Woodburn, Treasurer, submitted the following as his annual report:

Your Treasurer respectfully submits the following:

| | | |
|---|---|---|
| Received of Secretary Woolen, dues | | $593 00 |
| Paid balance due last year | $ 69 01 | |
| Paid interest on balance | 4 32 | |
| Paid J. G. Doughty, printing Transactions | 392 90 | |
| Paid Wright, Baker & Co., certificates | 3 50 | |
| Paid Todd & Carmichael, stationery | 7 80 | |
| Paid M. Meyer, janitor | 10 00 | |
| Paid C. W. Stagg, reporting | 46 75 | |
| Paid R. J. Bright, circulars, etc. | 9 75 | |
| Paid J. H. Holliday, advertising | 6 50 | |
| Paid L. D. Waterman, postage | 3 40 | |
| Paid G. V. Woolen, postage | 34 50 | |
| | | 588 43 |
| Balance in Treasury | | $ 4 57 |

JAMES H. WOODBURN, *Treasurer.*

The reports of the officers were referred to the Finance Committee.

Dr. Woolen submitted the following:

REPORT OF THE COMMITTEE ON PUBLICATION.

Your Committee would respectfully report, that they have issued 350 copies of the Transactions of last year at a cost of $392.90, and have sent a copy to each member who has paid his dues; also a copy to each of the leading journals of the country.

The physicians of Indianapolis very kindly furnished the engraving of the late Professor J. S. Bobbs, which is in the Transactions.

G. V. WOOLEN, *Chairman.*

On motion, the report was concurred in.

Dr. W. Lomax moved, that in consideration of the delicate health of the President he be requested to select such time as may best suit his convenience for the delivery of his annual address.

The motion prevailed, and the President announced that the address would be delivered at 3 P. M.

On motion of Dr. G. W. Butler, the reading of the minutes of the last annual session was dispensed with.

Dr. L. D. Waterman, from the Committee of Arrangements, submitted the following report:

Return free on certificate of Secretary—Indianapolis, Peru & Chicago; Bellefontaine & Indianapolis; Cincinnati & Indianapolis Junction.

Return for one-fifth fare, if the member buys a ticket in this city, on certificate of Secretary—Cincinnati & Indianapolis; Indianapolis & Lafayette; Indianapolis, Terre Haute & Vandalia; Indianapolis & St. Louis.

Louisville & Indianapolis sells excursion tickets only to members before coming. The arrangement was acceded to too late to be used.

Return only at full fare; refuse to make any reduction—Indiana Central; Indianapolis & Vincennes; Indianapolis, Bloomington & Western.

No other railroad arrangements made.

L. D. WATERMAN,
*Chairman Committee of Arrangements.*

The Finance Committee submitted the following report:

Your Committee would respectfully report that they have examined the Treasurer's account and find it correct.

T. M. STEVENS, *Chairman.*

The report was concurred in.

Dr. J. H. Woodburn moved a call of the special committees appointed at the last session for the purpose of ascertaining the number of papers prepared for consideration by the Society.

The motion prevailed.

The list being called by the Secretary, the following papers were reported:

On Diseases of the Eye—Dr. C. E. Wright, Indianapolis.
On Electricity as a Cause of Disease—Dr. Isaac Casselberry, of Evansville.
On Secondary Effects of Remedies—Dr. L. D. Waterman, of Indianapolis.
On Hydrocele—Dr. S. E. Munford, of Princeton.
On Médical Jurisprudence—Dr. T. M. Stevens, of Indianapolis.
On Lithotomy—Dr. R. E. Haughton, of Richmond.

The following voluntary papers were announced:

On the Anomalies of Refraction and Accommodation—Dr. J. Thompson, of Indianapolis.
On Expert Testimony—The conduct of witnesses in court, and the law which governs it—Dr. Wilson Hobbs, of Carthage.
Researches in Arsenical Poisoning—Dr. T. C. Van Nuys, of Evansville.
On Parotitis—Dr. L. J. Woollen, of Moorefield.
On Thoracentesis—Dr. N. Field, of Jacksonville.

The Congressional Districts were called for reports in the department of Medical Statistics, and the Fourth and Ninth Districts responded.

Dr. Wilson Hobbs moved to make the report of the special committee on Dr. Lomax's paper, appointed at the last annual session, the special order for the evening session. Carried.

Dr. J. A. Comingor, of Indianapolis, offered the following resolution:

*Resolved*, That this Society hold a general session in the forenoon of each day, and that there be three sections: First, of surgery and anatomy; second, the practice of medicine; and third, obstetrics and the diseases of women and children; and that the papers be appropriately distributed to those sections for discussion in the afternoon.

Dr. R. E. Haughton, of Richmond, moved to refer the proposition of Dr. Comingor to a committee, whose duty it shall be to inquire whether it is more expedient to make the proposed change or to add another day to the sessions of the Society, in order to admit of ample discussions in general session, said committee to report at the next annual session.

The motion prevailed.

Dr. W. T. Cady, of Lafayette, moved to make the report of the committee on Dr. Comingor's proposition, preferred business at the next annual session, which motion prevailed.

Dr. J. H. Woodburn announced the absence of Dr. B. Newland, of Bedford, the chairman of the Committee on Medical Ethics, and desired the society to fill the vacancy, that the committee might be prepared to act upon charges preferred against Dr. F. S. C. Grayston, of Huntington.

The Chair appointed Dr. Woodburn, chairman, and added Dr. G. W. Butler to the committee.

Dr. J. R. Wiest announced that the Committee on Prize Essay had no report to make, no business having come before it during the year.

Dr. S. E. Munford, of Princeton, read a paper on Hydrocele.

On motion of Dr. L. D. Waterman, the thanks of the Society were tendered Dr. Munford for his paper, and he was requested to submit it to the *Indiana Medical Journal* for publication.

The Society then adjourned till half-past one o'clock, P. M.

AFTERNOON SESSION.

The Society was called to order by the President, Dr. Ayres.

The following papers were read, and referred to the Committee on Publication :

By Dr. R. E. Haughton, of Richmond—On the Pathology of Malignant and Semi-Malignant Growths.

By Dr. L. J. Woollen, of Moorefield—On an epidemic of Parotitis (mumps) in Switzerland county.

By the President—His annual address.

By Dr. T. C. Van Nuys, of Evansville—Researches in Arsenical Poisoning.

By Dr. J. Thompson, of Indianapolis—On the Anomalies of Refraction and Accommodation.

By Dr. Wilson Hobbs, of Carthage—On Expert Testimony, the conduct of witnesses in court, and the law which governs it.

The last named paper elicited discussion by Drs. Waterman, Boyd and offit, after which

Dr. Haughton moved that a committee of three be appointed to take into consideration the means to be used for the purpose of securing a definition by the laws of the State, of what constitutes a physician or a medical expert—the committee to report at this session. The motion prevailed.

The Chair appointed Dr. R. E. Haughton as chairman of the committee, and upon nomination, Drs. L. D. Waterman and Wilson Hobbs were appointed as the remaining members.

The Society then adjourned till 8 o'clock, P. M.

EVENING SESSION.

The Society was called to order by the President.

Dr. Lomax, from a special committee appointed at the last annual session on the objects and duties of the Indiana State Medical Society, submitted a report signed by all the committee, and embracing the following resolutions :

1. *Resolved*, That it is the duty of the Society to at once adopt such measures as may tend most directly to bring the entire profession of the State into one harmonious body, for the purpose of carrying out the objects for which the Society was organized.

2. *Resolved*, That a committee of three from each Congressional District in the State be appointed, to confer with such County Societies as may exist in their respective districts, and secure as far as possible the adoption by them of a uniform constitution auxiliary to the State Society, and also the incorporation of such local Societies under the laws of the State, and in those counties where no such Medical Societies exist, use every effort to effect their organization.

3. *Resolved*, That such District Committees shall report to the Secretary of the State Society every local Society that shall have been thus incorporated, as soon as such incorporation shall have been perfected, with the names and postoffice addresses of all its officers and members, and when twelve such County Societies shall have been reported, the Secretary of the State Society shall at once notify such corporate County Societies to elect delegates in the ratio of representation prescribed by Section I. of Article IV. of the Constitution, to meet in Convention at such time and place as he, the Secretary of the State Society, shall designate, to organize and incorporate the State Society under the law of the State, and that such delegation, when thus assembled, shall proceed to elect their officers and adopt a Constitution, and have the same legally recorded, and do all acts and things necessary to secure to the State Society all the powers, privileges and immunities of a corporate body.

4. *Resolved*, That a committee of five be appointed to prepare a suitable form of constitution for the organization of County Societies, to be presented for approval at the present meeting, and that such forms be furnished the committees on organization for use in aiding the organization of such societies.

5. *Resolved*, That an assessment of two dollars be levied on each and every member of County Societies for the use of the State Society.

On motion, the resolutions were considered *seriatim*.

The first and second resolutions were adopted.

Dr. R. N. Todd moved to amend the third resolution so as to instruct the contemplated convention of delegates to frame the law so that the present permanent membership of the State Medical Society in good standing shall be retained in the proposed incorporation.

The resolution and amendment were made the subject of a free discussion, participated in by Drs. Waterman, Lomax, Todd, Boyd, McClellan, Haughton, Hobbs, Van Voris, Wollen, and Moffitt.

The amendment prevailed, and the third resolution, so amended, was adopted.

The fourth and fifth resolutions were adopted.

The Chair appointed as the committee to form the constitution of County Societies, Drs. W. Lomax, James H. Woodburn, Wilson Hobbs, William Scott, G. W. H. Kemper, and John Moffitt.

The Society then adjourned till 8 A. M., 22nd.

---

SECOND DAY—MORNING SESSION.

The Society came to order at eight o'clock, the President in the chair.

The President appointed the following standing committees:

On Prize Essays—Drs. T. Parvin, of Indianapolis; S. E. Munford, of Princeton, and G. W. H. Kemper, of Muncie.

On Medical Ethics—Drs. V. Kersey, of Richmond; F. J. Van Voris, of Indianapolis, and J. S. Gregg, of Fort Wayne.

On Arrangements—Drs. A. W. Davis, E. A. Tomlinson, and F. J. Van Voris, all of Indianapolis.

On Finance—Drs. W. B. Lyons, of Huntington; W. F. Cady, of Lafayette, and Isaac Casselberry, of Evansville.

On Publication—Drs. G. V. Woolen, T. M. Stevens, and J. A. Comingor, all of Indianapolis.

On Nominations—Drs. C. S. Arthur, of Portland; Isaac Casselberry, of Evansville; W. Lowry, of Marion; T. M. Stevens, of Indianapolis, and Edwin Hadley, of Richmond.

The Committee on Credentials reported correct, the credentials of Drs. F. J. Spillman, of the Rush Medical Society; W. H. Bell, of Brainard Medical Society, and George Sutton, of the Dearborn County Medical Society.

The Committee on Membership at Large reported in favor of the admission to membership of Drs. James O. Ward, of Peru, and Simeon S. Marsh, of Reserve.

Both reports were concurred in, and the persons therein named elected to membership.

The Committee on Medical Statistics was, on motion, continued for the ensuing year. They are as follows :

First District—Dr. D. Morgan, of Evansville.
Second District—Dr. Wm. A. Clapp, of New Albany.
Third District—Dr. A. G. Boynton, of Elizabethtown.
Fourth District—Dr. S. M. Martin, of Greenfield.
Fifth District—Dr. Henry G. Todd, of Danville.
Sixth District—Dr. T. H. Rice.
Seventh District—Dr. T. J. Adams, of North Salem.
Eighth District—Dr. W. K. Mavity, of Kokomo.
Ninth District—Dr. N. H. Canaday, of Knightstown.
Tenth District—Dr. H. D. Wood, of Angola.
Eleventh District—Dr. Lewis Humphreys, of South Bend.

Dr. Thaddeus M. Stevens, of Indianapolis, read a paper on Legal Medicine. The paper advocated the formation of a corps of medical experts, with a view to the relief of the profession in general from the embarrassment of examination in court. It also advised that when physicians are called to testify on questions of sanity, they confine their answers to the simple statement that the party is sane or insane, without attempting to define the particular grade of insanity in any case.

Dr. John Moffit, of Rushville, said the advice of the essayist would do very well if courts and lawyers would be put off by such an answer, but that the witness was sure to be probed to the very bottom of his knowledge on cross-examination, and must either show himself thoroughly acquainted with the subject or leave the stand in disgrace. The only way for the medicine man to meet the difficulty is to understand the matter whereof he speaks, or say he knows nothing about it before he is called upon the stand.

The paper was then referred to the Committee on Publication.

Dr. C. E. Wright, of Indianapolis, read a paper on Diseases of the Eye and Ear. Referred to the Committee on Publication.

Dr. R. E. Haughton, of Richmond, from a special committee, submitted the following report :

The Committee to whom was referred the subject of petitioning the Legislature, beg leave respectfully to report the following resolution :

*Resolved*, That a committee of three be appointed to petition the Legislature of Indiana for the passage of an anatomical law, embodying such rights and protection, as well as such wise restraints as shall prevent abuse in the study of anatomy, pathology and surgery as shall not subject the profession to odium, but which will enable its members successfully to qualify themselves for the practice of their profession, and fill the position of medical witnesses and experts in a satisfactory and honorable manner, thus facilitating the administration of justice and promoting the interests of science.

(Signed) R. E. Haughton.
Wilson Hobbs.
L. D. Waterman.

The report was concurred in, and the resolution adopted.

Dr. N. Field, of Jeffersonville, announced as follows, the death of a prominent member of the profession :

It is my painful duty to announce the death, after a painful illness, of Dr. Robert Curran, of Jeffersonville. The whole history of the deceased illustrates the purity of his private character and the dignity of the medical profession. He was a man noted for his industry and application to business. He was an ardent admirer of the profession which he had made the business of the principal part of his life, and in every way he exemplified and adorned the profession of which he was a distinguished member. I beg leave to offer the following resolutions:

*Resolved*, That in the death of Dr. Robert Curran, of Jeffersonville, the medical profession has lost one of its brightest ornaments and the Society a valued member—one who never for a moment compromised its honor or his own professional self-respect, but who exemplified in his own life of labor and usefulness its dignity and respectability.

*Resolved*, That the Indiana State Medical Society tender to the family of the deceased their sincere condolence in their bereavement.

*Resolved*, That these resolutions be published in the Transactions of this Society, with a brief biographical sketch of the deceased.

Dr. G. W. Mears, of Indianapolis, seconded the resolutions as follows:

As a former colleague of Dr. Curran, it would give me great pleasure if I were able to speak at some length in reference to his character as a man and as a member of our profession, but in consequence of my feebleness I shall only express my hearty concurrence in what has been said in regard to him and in the sentiment embodied in the resolutions. He was an excellent and an able man, and gave evidence of his strong attachment to the profession which he had chosen and followed for a great many years most successfully.

The resolutions were then adopted by a rising vote.

Dr. Field presented to the Society a biographical sketch of Dr. Curran, written by his son-in-law, Dr. Seymour, and also a professional essay written by the deceased on the "Nosology of the Diseases of Clark County."

The biographical sketch was referred to the Committee on Publication, and on motion of Dr. Waterman, Dr. D. Clarke, of Richmond, was requested to read the medical essay during the afternoon session.

Dr. T. Parvin presented a communication from the Committee on Medical Education of the National Medical Association.

Dr. Waterman moved that the communication be referred to a special committee of three, and that their report be made the special order of business for four o'clock, P. M. The motion prevailed, and Drs. T. Parvin, Isaac Casselberry and W. Lomax were appointed said committee.

Dr. T. M. Stevens, from the Standing Committee on Nominations, submitted the following report, which was concurred in:

For President, Joel Pennington, of Milton.
For Vice President, R. E. Haughton, of Richmond.
For Secretary, G. V. Woolen, of Indianapolis.
For Assistant Secretary, W. J. Elstun, of Indianapolis.
For Treasurer, J. H. Woodburn, of Indianapolis.
For Librarian, A. W. Davis, of Indianapolis.
For Delegates to National Medical Association—Drs. W. Lomax, E. H. Crispen, J. Helm of Peru, H. V. Passage, G. W. Mears, S. B. Harvey, Edwin Hadley, John Comingor, Gregg of Fort Wayne, B. S. Woodworth, Wm. F. Cady, Thos. C. VanNuys, S. E. Munford, Wm. R. Davidson, N. Field, George Sutton, A. G. Preston, I. F. Beckner, I. C. Walker, A. M. Vickrey, H. P. Ayres, Isaac Johnston, R. B. Jessup, and V. Kersey.

Delegates to the State Medical Society of Kentucky—Drs. Isaac Casselberry, and Thos. C. VanNuys.

Delegates to Ohio State Medical Society—Drs. C. S. Arthur, T. Parvin, and L. D. Waterman.

Delegates to Illinois State Medical Society—Drs. R. N. Todd, T. M. Stevens, and G. W. Mears.

Delegates to Michigan State Medical Society—Drs. H. P. Ayres, and F. S. C. Grayston.

Dr. N. Field, of Jeffersonville, read a paper on Theoracentesis, which was discussed by Drs. R. N. Todd, of Indianapolis, J. I. Rooker, of Castleton, H. V. Passage, of Peru, and R. E. Haughton, of Richmond.

Dr. R. E. Haughton read a paper on Lithotomy, which was discussed by Drs. Comingor, Boyd, Waterman, and Haughton.

The Society then adjourned till 1½ P. M.

AFTERNOON SESSION.

The Society was called to order by the President.

Dr. Isaac Casselberry, of Evansville, read a paper on Electricity as a Cause of Disease, which was referred to the Committee on Publication.

Dr. Woodburn, from the Committee on Ethics, submitted the following report, which, on motion of Dr. Waterman, was concurred in:

Your Committee have examined the charges preferred last year against Dr. Grayston for violating the code of ethics, and report that, after hearing the testimony, we find him *not guilty*.

J. H. Woodburn,

D. H. Oliver,

D. W. Butler, } *Committee.*

Dr. Woodburn, from the Committee on Ethics, reported in favor of the expulsion of J. T. Brenton, M.D., of Edinburg, for having signed the following certificate:

Edinburg, Ind., Aug. 28, 1871.

This is to certify, that I have used Brown's Expectorant in my family since its first introduction. It has never failed to give entire satisfaction. My wife is subject to bronchitis, and I have no remedy equal to Brown's Expectorant. I recommend it as a safe and reliable medicine. J. T. Brenton, M.D.

Dr. Comingor moved to refer the case back to the Committee, with instructions to cite Dr. Brenton to trial, and to report at the next annual session.

Dr. G. Sutton, of Aurora, offered for adoption the following resolution, which was adopted:

*Resolved*, That we recommend to the Board of Trustees of the Indiana University that they introduce as soon as practicable, either into the scientific or medical department, a course of studies embracing Comparative Anatomy, Comparative Physiology, and Comparative Pathology—branches of science arising from the progress of knowledge, becoming daily of great practical importance in understanding the paleontology of our country, the zoology of our State, and the diseases which are producing such destructive ravages amongst our domestic animals.

Dr. T. M. Stevens moved that the Secretary be instructed to transmit a certified copy of the resolution to the trustees of the Indiana State University. Carried.

Dr. L. D. Waterman, of Indianapolis, read a paper on Secondary Effects of Remedies, which, after discussion, was referred to the Committee on Publication.

Dr. T. Parvin, from a special committee, submitted the following report:

The Committee to whom was referred the communication from the Special Committee on Medical Education of the American Medical Association, beg leave to present the following report:

This communication embraces, as inviting the action of the Indiana State Medical Society, first, resolutions relating to the admission of persons to the practice of medicine, providing for the examination of such, for the compensation of examiners, and affixing a penalty for neglecting such examination; and, second, a resolution relating to the admission of those desiring to study medicine, to the offices of physicians or to medical colleges.

We believe the elevation of the profession must arise from a force within itself, that all exterior ends are merely secondary. And we should believe that a most important step towards their elevation will be taken when the medical profession shall determine whom they will recognize as qualified students and qualified practitioners. We therefore recommend the adoption of the several resolutions; and if their adoption should be made with hearty unanimity, or even an approximation to it, we believe the good health will be neither tardy nor doubtful.

Should their State Society take such action, it places itself in the way of progress in medical education in this country, for only four other State Societies have this action.

T. Parvin,<br>

I. Casselberry, } *Committee.*<br>

W. Lomax,

The resolutions referred to by the Committee and embodied in the report were as follows:

*Resolved*, That each State and local medical society be requested to provide as a permanent part of its organization a Board of Censors for determining the educational qualifications of such young men who propose to commence the study of medicine, and that no member of such societies be permitted to receive a student into his office until such student presents a certificate of proper preliminary education from the censors appointed for that purpose, or a degree from some literary college of known good standing.

*Resolved*, That this association earnestly requests each State Medical Society to appoint annually one or more Boards of Examiners, composed of five thoroughly competent members, whose duty it shall be to meet, at suitable times and places, for the examination of all persons, whether graduates of colleges or not, who propose to enter upon the practice of medicine in their respective States, except such as have been previously examined and licensed by a similar Board in some other State.

*Resolved*, That each State Medical Society be requested to make such regulations concerning the pay of the Board of Examiners, and the fee to be charged for a license to practice, and that the former shall in no case depend on the amount received from the latter.

*Resolved*, That this State Medical Society be requested to require its Examining Board or Boards to exact of every applicant for examination adequate proof that he has a proper general education, is twenty-one years of age, and has pursued the study of medicine three full years, one-half of which time shall have been in some regularly organized medical college, whose curriculum embraces adequate facilities for didactic, demonstrative and hospital clinical instruction.

The first, second and third resolutions were adopted. The fourth resolution was lost.

Dr. D. W. Butler presented the following resolutions, which were unanimously adopted:

*Resolved*, 1. That the thanks of this Society are due, and are hereby tendered to President Ayers for the able manner in which he has presided over and conducted the deliberations of this body.

2. To the Trustees of the Indiana Medical College for the use of their hall.

3. To the railroads who have so kindly accommodated the delegates and members with reduced fare.

4. To the press of the city for the daily reports of our proceedings, and to Drs. Woolen and Elstun, the Secretary and Assistant Secretary, for their efficient services during the year.

Dr. W. Lomax, of Marion, from a special committee, reported a form of constitution for County Medical Societies. The constitution reported, which was that of the Grant County Medical Society, was adopted as a whole.

Dr. F. J. Van Voris, of Indianapolis, offered the following resolution, which was adopted:

*Resolved*, That this Society recommend to the Legislature of Indiana to adopt and incorporate the Indiana Medical Society as a branch of the Indiana State University, with all the powers, rights and privileges thereunto pertaining.

The Society then adjourned till eight o'clock, P. M.

EVENING SESSION.

The Society was called to order by the President.

The Secretary read a report by Dr. J. M. C. Adams, of Frankfort, on the prevailing diseases and their treatment, of the Seventh Congressional District.

Dr. Isaac Casselberry moved the reconsideration of the vote taken this afternoon on the resolution recommended by the National Medical Association—which motion prevailed.

Dr. Casselberry moved to lay the resolution on the table. Carried.

On motion, the paper by Robert Curran, M.D., entitled Nosology of the Diseases which have prevailed in Clarke County, Indiana, since A. D. 1833, was read by its title, and referred to the Committee on Publication.

Dr. L. D. Waterman offered the following resolution:

*Resolved*, That Professor John A. Murphy, of Cincinnati, O., is hereby elected an honorary member of this Society.

The Committee on Membership at Large reported in favor of the admission to membership of Dr. C. H. Smith, of Richland; Dr. O. W. Brownback, and Dr. E. C. Locke, of Nobleville; Dr. James P. Orr, of Andersonville; and Dr. B. Baker, of Alexandria. All of whom were thereupon elected to membership.

The Committee on Credentials reported correct the credentials of Drs. S. J. Shirley and S. C. Johnson, of the Howard County Medical Society. The report was concurred in.

Dr. Lomax moved that Dr. L. D. Waterman be added, as chairman, to the Committee appointed to present the matter of a registration law to the Legislature, and that the Committee be continued for the ensuing year. Carried.

Dr. T. B. Harvey moved that the action continuing the Committee on Medical Statistics be abrogated, that Committee be discharged, and that a new Committee be appointed, with Dr. G. Sutton, of Aurora, as Chairman. Carried.

Dr. T. M. Stevens moved that Dr. Sutton, Chairman, and Dr. Woolen, Secretary of the Society, be empowered to appoint the members of the Committee. Carried.

The Secretary read the following list of Special Committees to prepare papers for the next annual session:

On Cerebro-Spinal Meningitis, by Dr. William F. Cady, of LaFayette.

On the Correlation of Physical and Vital Forces, by R. E. Haughton, of Richmond.

Experimental Researches into the Poisonous and Chemical Properties of Atropia, by Dr. Thomas C. VanNuys, of Evansville.

On the Toxicological Action of and Examination for the Alkaloid, by Thad. M. Stevens, M.D., of Indianapolis.

On the Secondary Effects of Gunshot Wounds, by Dr. J. S. Gregg, of Fort Wayne.

On Cerebral Meningitis, as it prevailed in Montgomery county, Indiana, during the Spring of 1872, by Samuel G. Irwin, M.D., of Crawfordsville, Indiana.

On Malignant Tumors, by Dr. L. D. Waterman, of Indianapolis.

On Surgical Cases which have Fallen under my Observation, by Dr. J. S. McClelland, of Crawfordsville.

On Chemico-Legal Investigations, by Dr. P. McNab, of West Newton.

On Cholera Infantum, by Dr. G. N. Duzan, of Zionsville.

On Diseases of the Eye and Ear, by Dr. C. E. Wright, of Indianapolis.

On such subject as he may choose, by Dr. J. W. Hervey, of Oakland.

On motion of Dr. Waterman, the Secretary was authorized to add such further essays to the list as should be handed to him in time for publication in the printed proceedings of the session.

The Society then adjourned till the third Tuesday of May, 1873.

